# Supplementary material for: Immune activation during broadly neutralizing antibody-mediated HIV suppression prior to post-intervention control
Source: bioRxiv. 2026 Jun 2:2026.05.29.728863. Preprint. [Version 1] doi: 10.64898/2026.05.29.728863 (PMC13251981; doi:10.64898/2026.05.29.728863)
Supplement: Supplement 1 [file media-1.pdf]

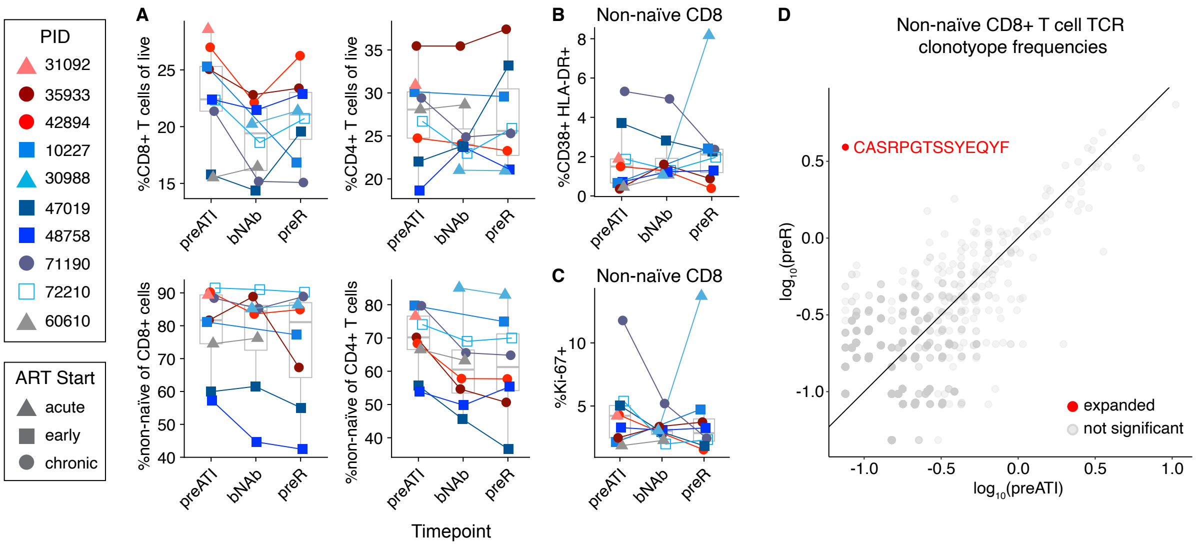

Supp. Fig. 1: T cell frequency and activation are unchanged under bNAb-mediated HIV suppression. (A) Frequencies of total and non-naïve CD4+ and CD8+ T cells at the *preATI*, *bNAb*, and *preR* timepoints. (B) Frequency of activated (CD38+HLA-DR+) non-naïve CD8+ T cells at the *preATI*, *bNAb*, and *preR* timepoints. (C) Frequency of proliferative (Ki-67+) non-naïve CD8+ T cells at the *preATI*, *bNAb*, and *preR* timepoints. (D) An example plot showing non-naïve CD8+ T cell TCR clonotype frequencies from 10x scRNA/TCRseq data at the *preR* (y-axis) versus *preATI* (x-axis) timepoints (1 clonotype significantly expanded in PID 30988 at *preR* relative to *preATI*). For A-C: data were compared using linear mixed effects models with Benjamini-Hochberg correction; no comparisons were statistically significant ($p_{adj}<0.05$). For D: significant expansion was defined as $p_{adj} <0.1$ and $\log_2$ fold-change ≥1 in more than 50 of 100 repeats. Box plots indicate median (line) and interquartile range (boxes).

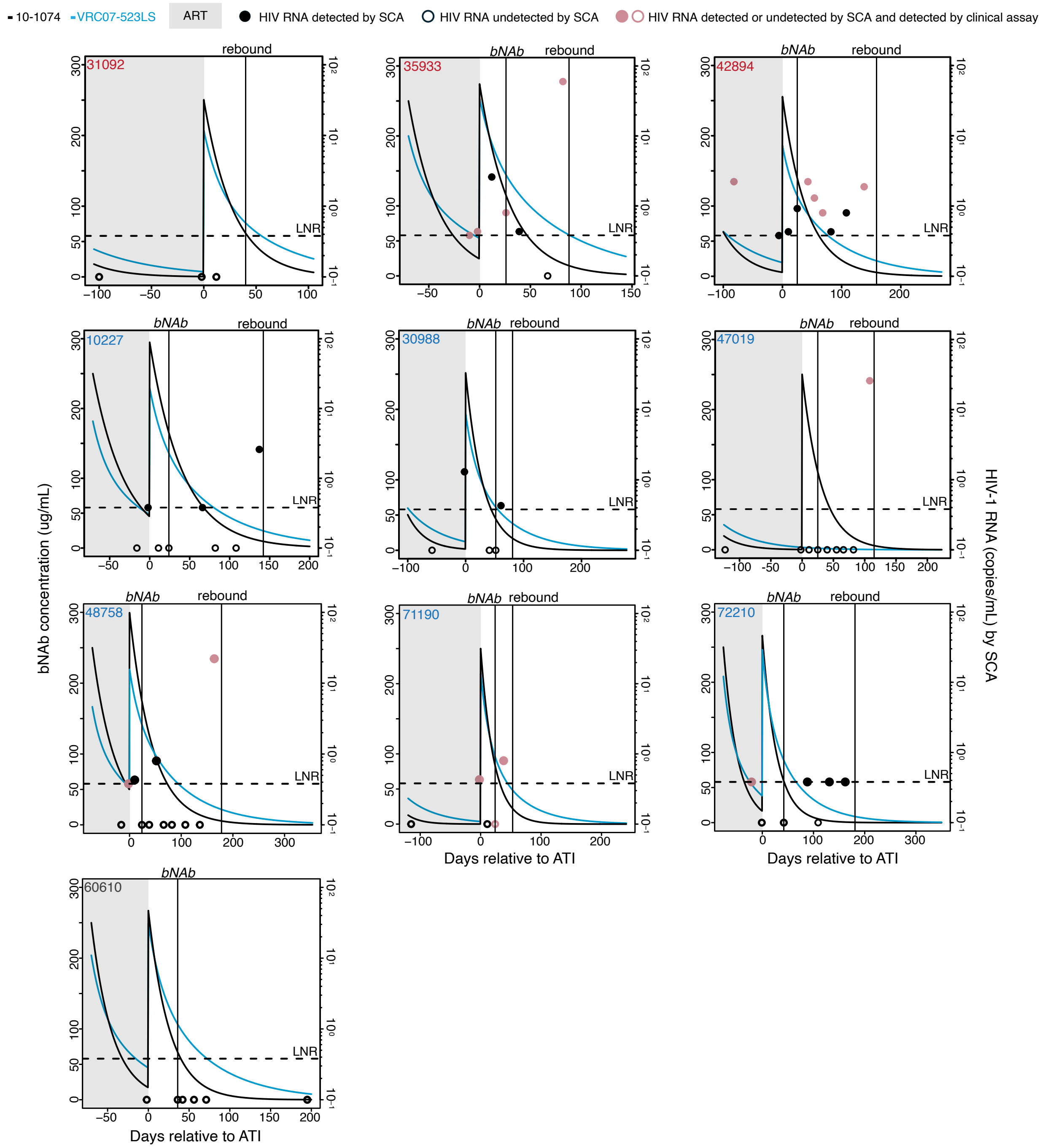

Supp. Fig. 2: Minimal residual plasma viremia detectable during bNAb-mediated HIV suppression. bNAb levels (10-1074 [black] and VRC07-523LS [blue]) and HIV-1 RNA levels (circles) by single copy assay for each participant at the indicated day relative to ATI (day 0). The *bNAb* timepoint and time of rebound are indicated with a solid vertical line for each participant. For the viral loads, black indicates HIV-1 RNA was not detected by clinical assay at that timepoint and rose indicates HIV-1 RNA was detected by clinical assay. Solid circles indicate HIV-1 RNA was detected by single copy assay at that timepoint versus empty circles indicate HIV-1 RNA was not detected by single copy assay. A shaded grey background indicates ART suppression.

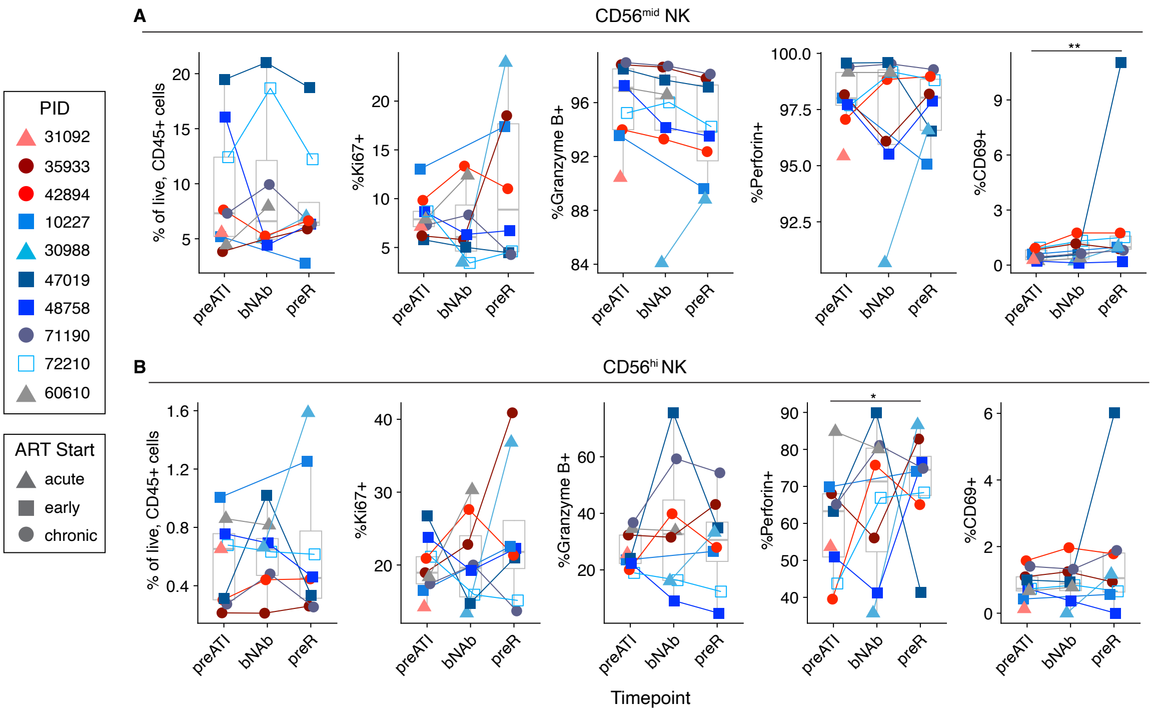

Supp. Fig. 3: Additional innate immune activation markers. Ki-67+, Granzyme B (GzB)+, Perforin+, CD25+, and CD69+ of (A) CD56^{mid} or (B) CD56^{hi} NK cells at the *preATI*, *bNAb*, and *preR* timepoints. Data were compared using linear mixed effects models with Benjamini-Hochberg correction. * = $p_{adj}<0.05$, ** = $p_{adj}< 0.01$. Box plots indicate median (line) and interquartile range (boxes).

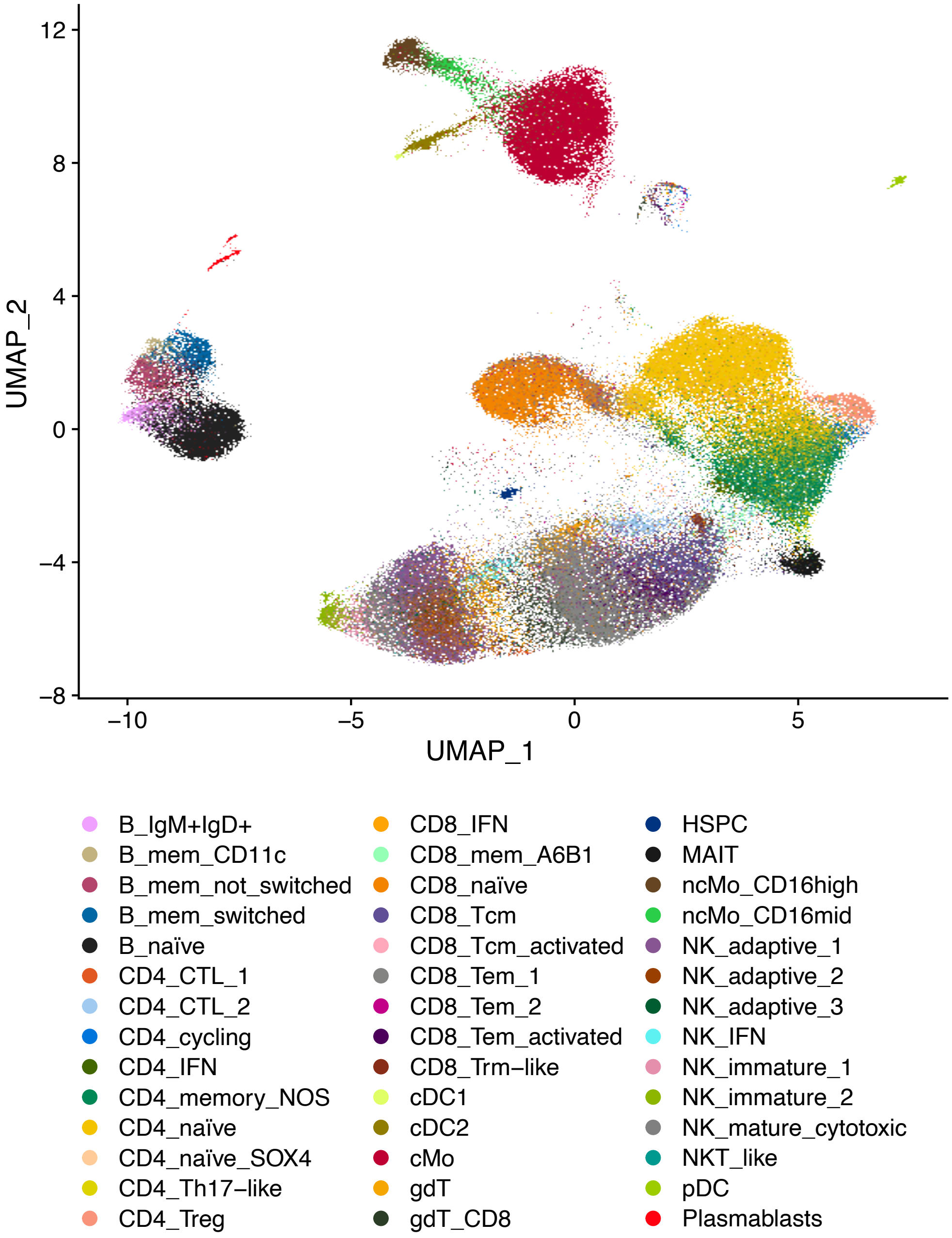

Supp. Fig. 4: UMAP of all 42 cell type subclusters identified using CITEseq.

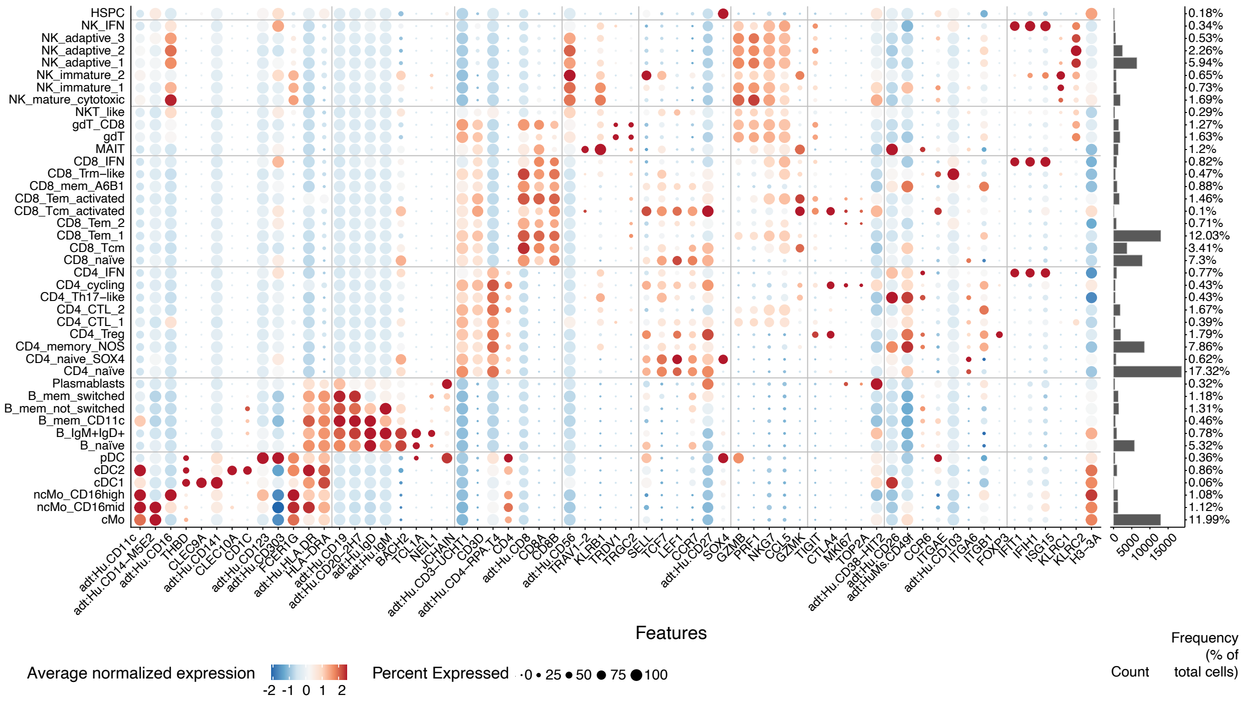

Supp. Fig. 5: Gene transcript and protein expression data used to identify CITEseq cell type clusters. Abbreviations: hematopoietic stem cell (HSPC), natural kill cell (NK), interferon (IFN), γδ T cell (gdT), mucosal-associated invariant T cells (MAIT), T resident memory (Trm), memory (mem), effector memory (em), central memory (cm), cytotoxic lymphocyte (CTL), not otherwise specified (NOS), plasmacytoid dendritic cell (pDC), conventional dendritic cell (cDC), non-classical monocyte (ncMO), classical monocyte (cMo).

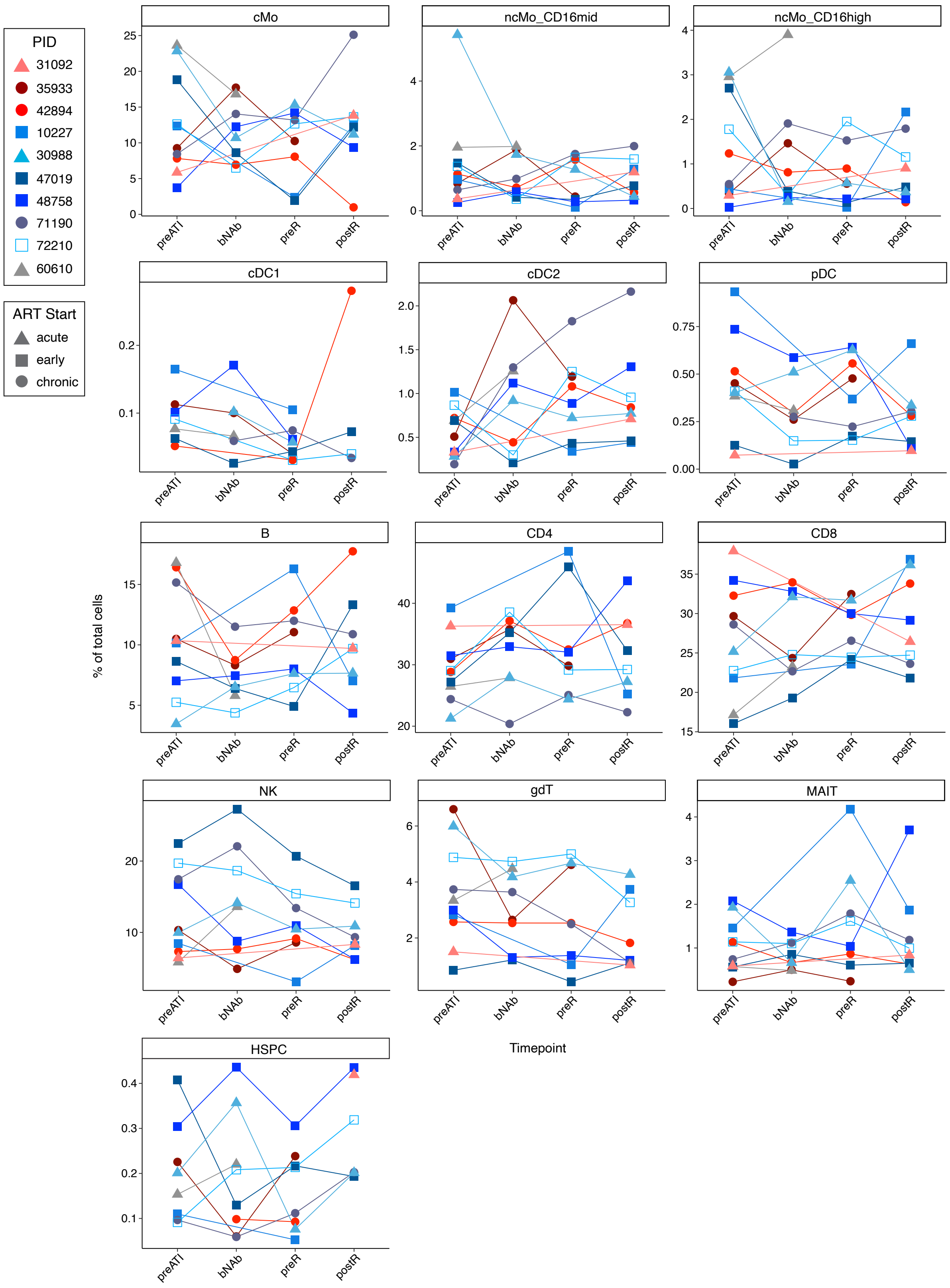

Supp. Fig. 6: Frequencies of landmark populations under bNAb-mediated HIV suppression by CITEseq. Frequencies are of total cells.

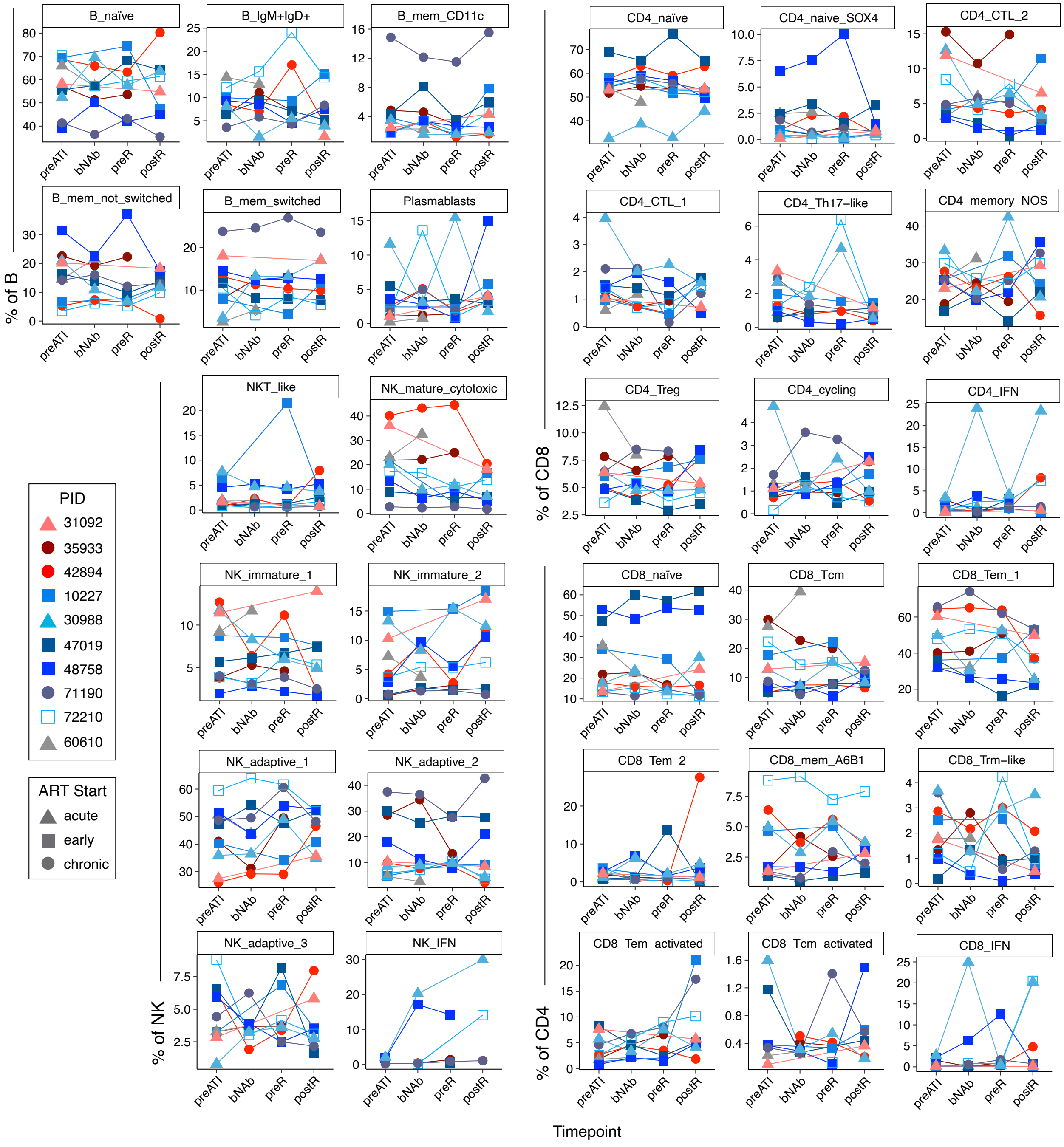

Supp. Fig. 7: Frequencies of cell type subclusters under bNAb-mediated HIV suppression by CITEseq. Frequencies are of parent population (B cells, NK cells, CD4+ T cells, CD8+ T cells).

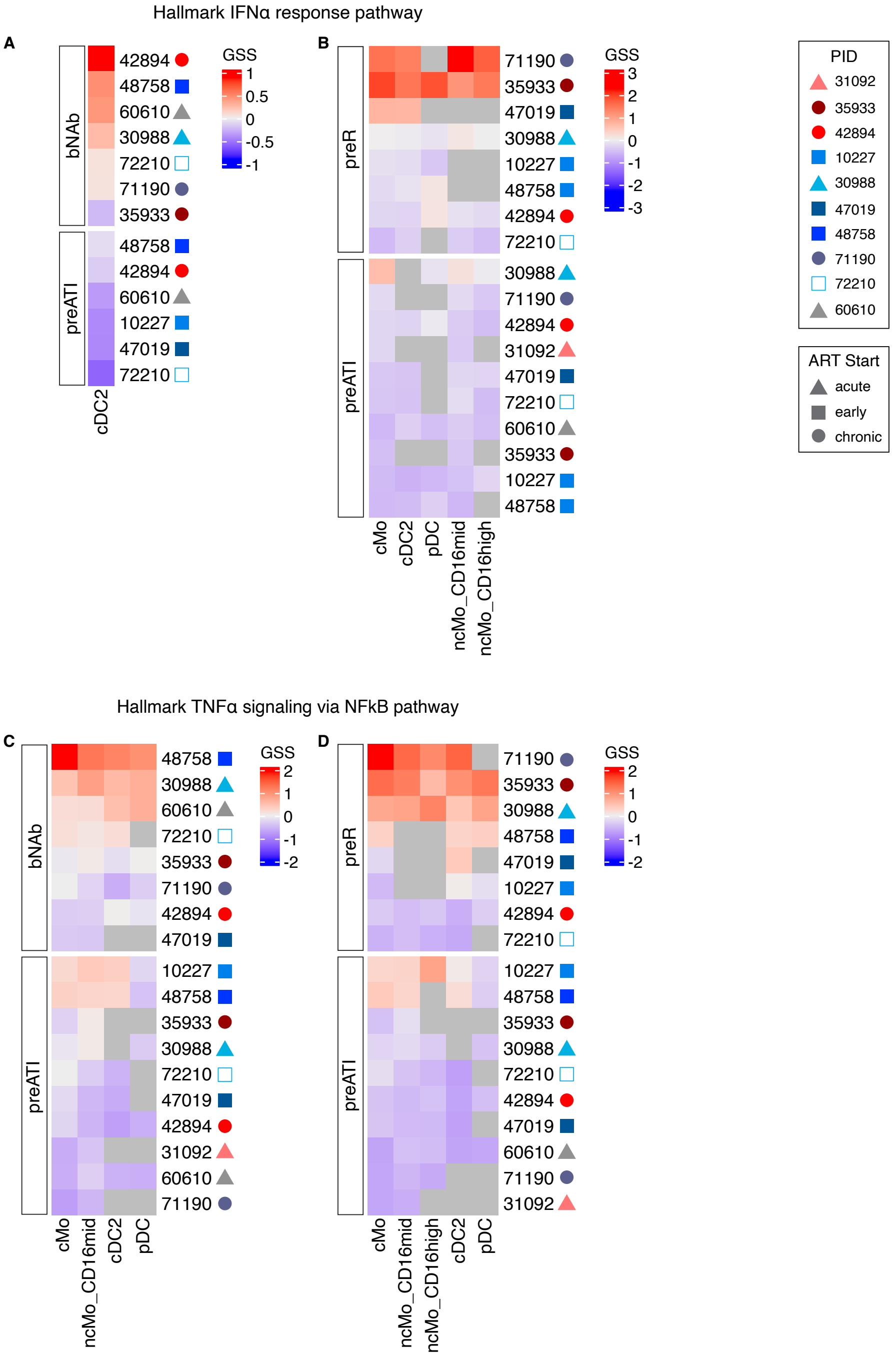

Supp. Fig. 8: IFNα and TNFα signaling gene signature scores by participant for between timepoint comparisons. Gene signature scores (GSS) for the Hallmark Interferon (IFN)α response and Hallmark TNFα signaling via NFκB pathways for each indicated cell type by participant at the (A, C) *bNAb* and *preATI* and (B, D) *preR* and *preATI* timepoints. Gene signature score reflects the mean of the Z-scored expression of leading-edge genes of the relevant pathway in the indicated sample/cell type. Grey boxes indicate no data.

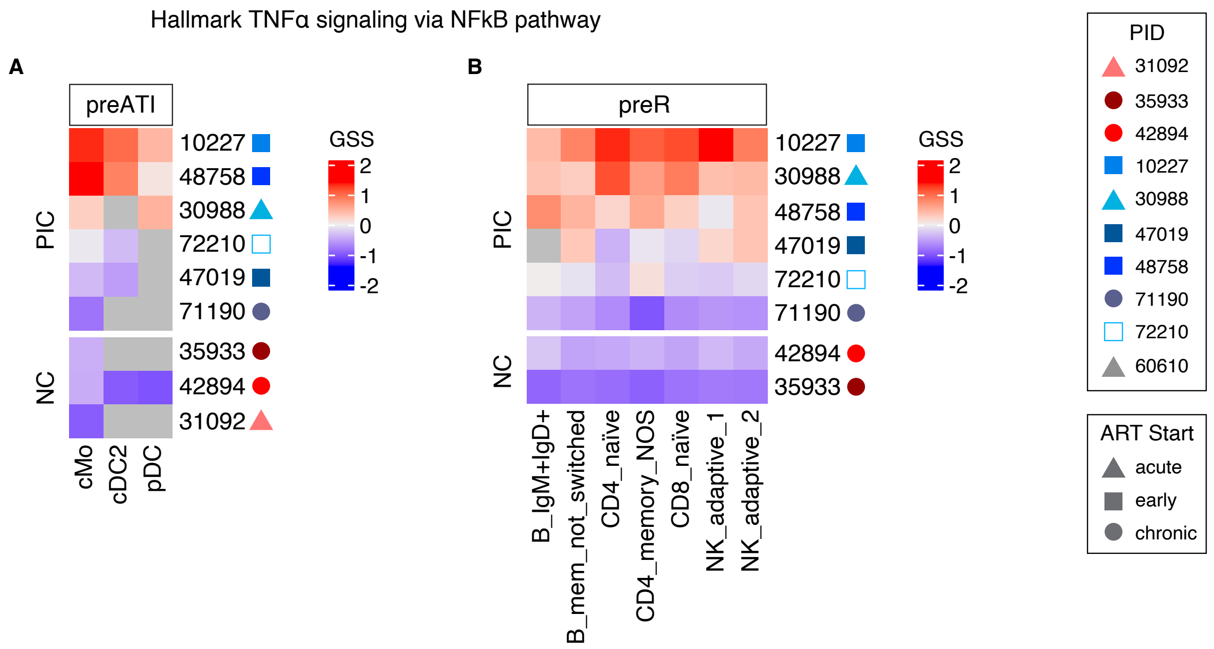

Supp. Fig. 9: TNFα signaling gene signature scores by participant for controller versus non-controller comparisons. Gene signature scores (GSS) for each indicated cell type by participant at the (A) *preATI* and (B) *preR* timepoints. Gene signature score reflects the mean of the Z-scored expression of leading-edge genes of the Hallmark TNFα signaling via NFκB pathway in the indicated sample/cell type. Grey boxes indicate no data. NC = non-controller, PIC = post-intervention controller.
